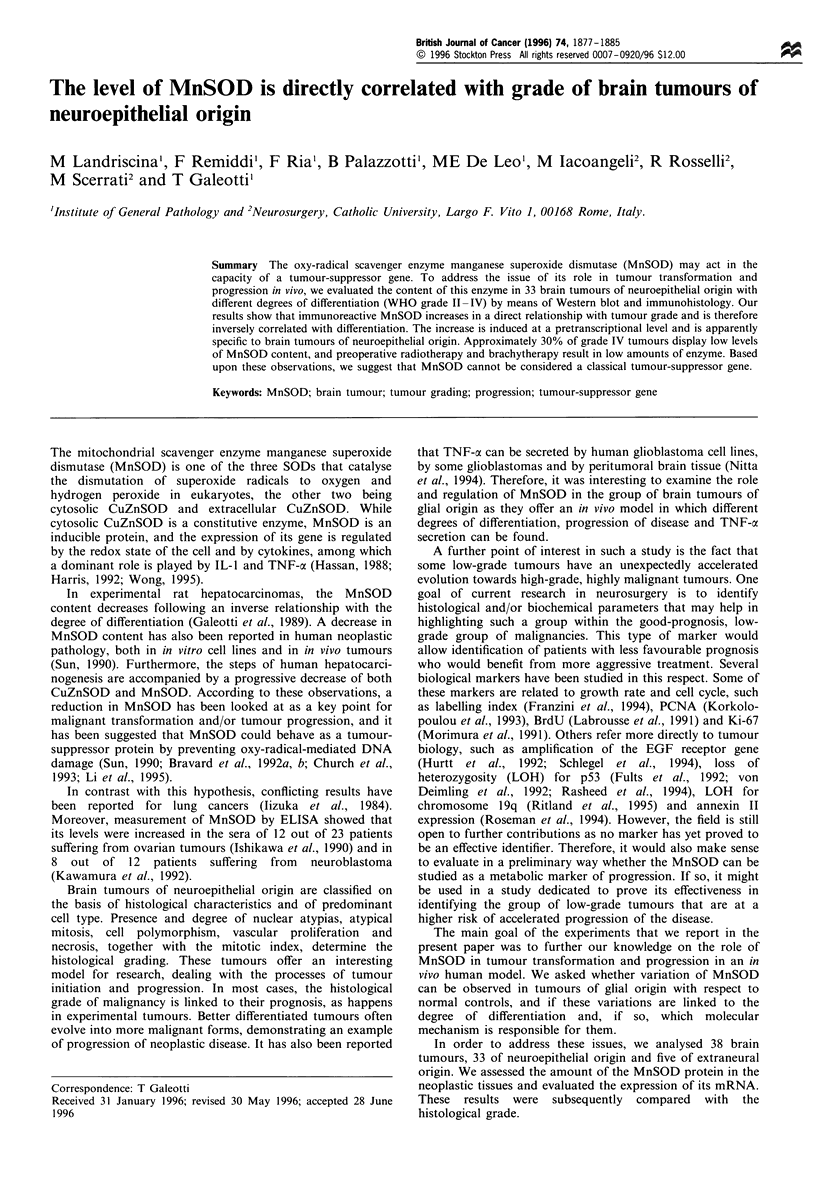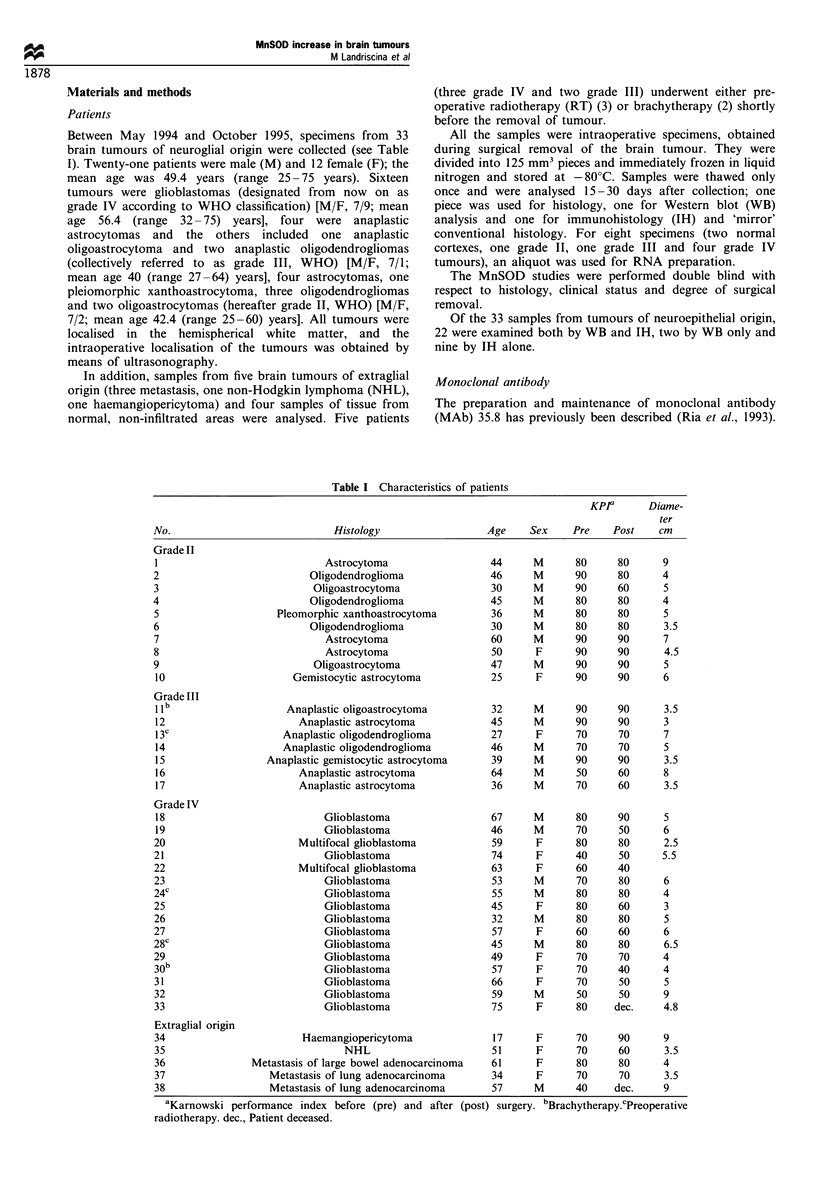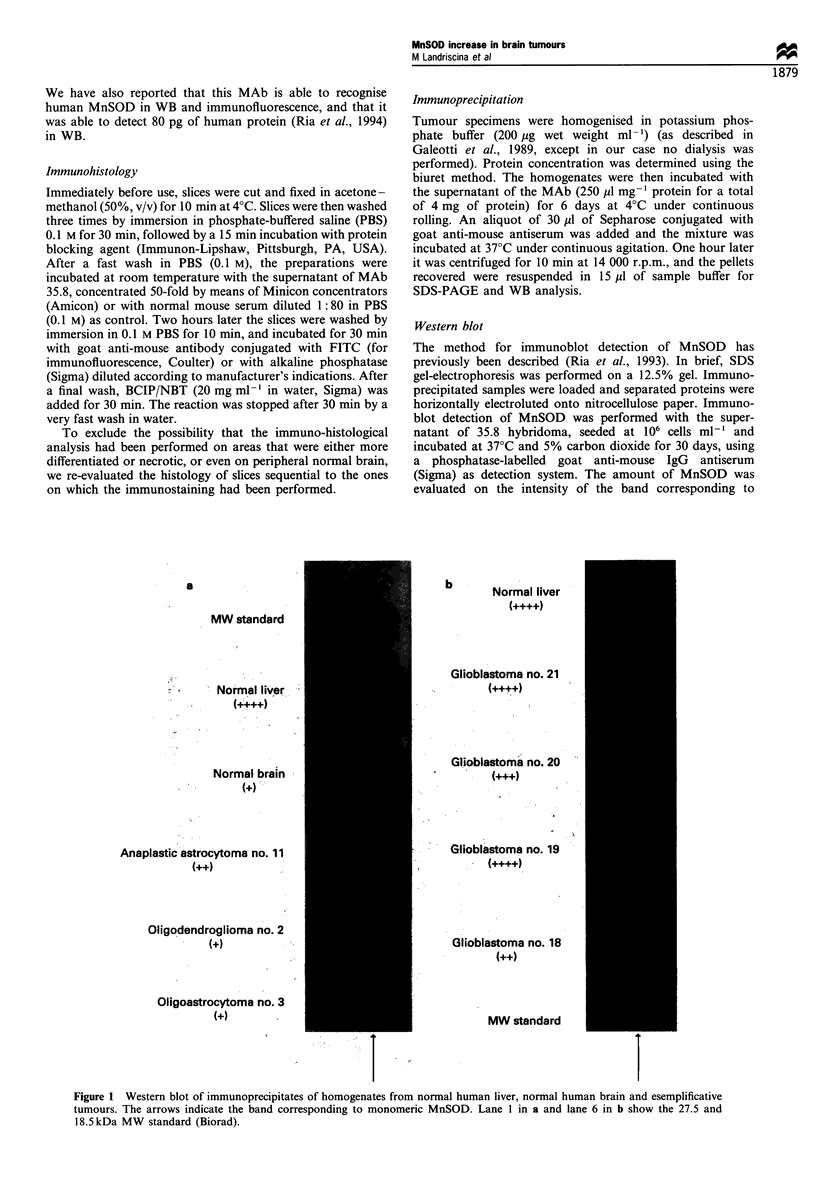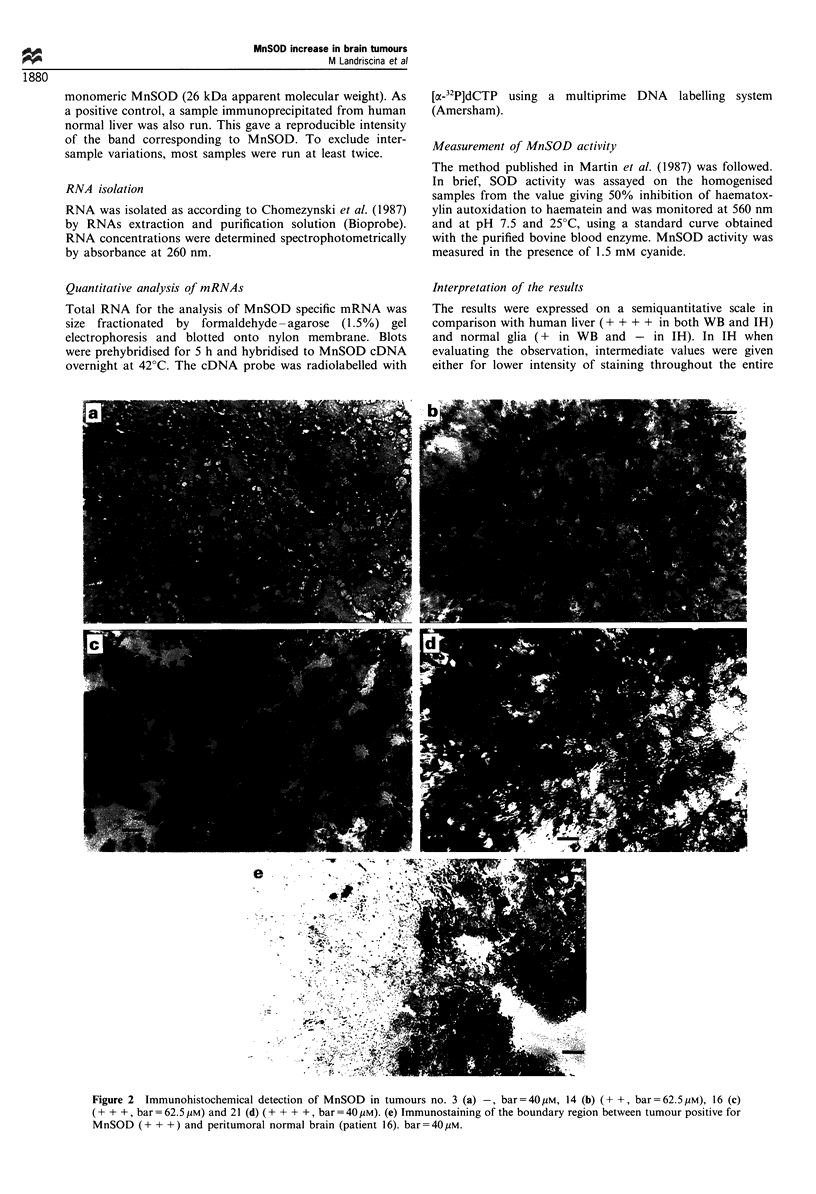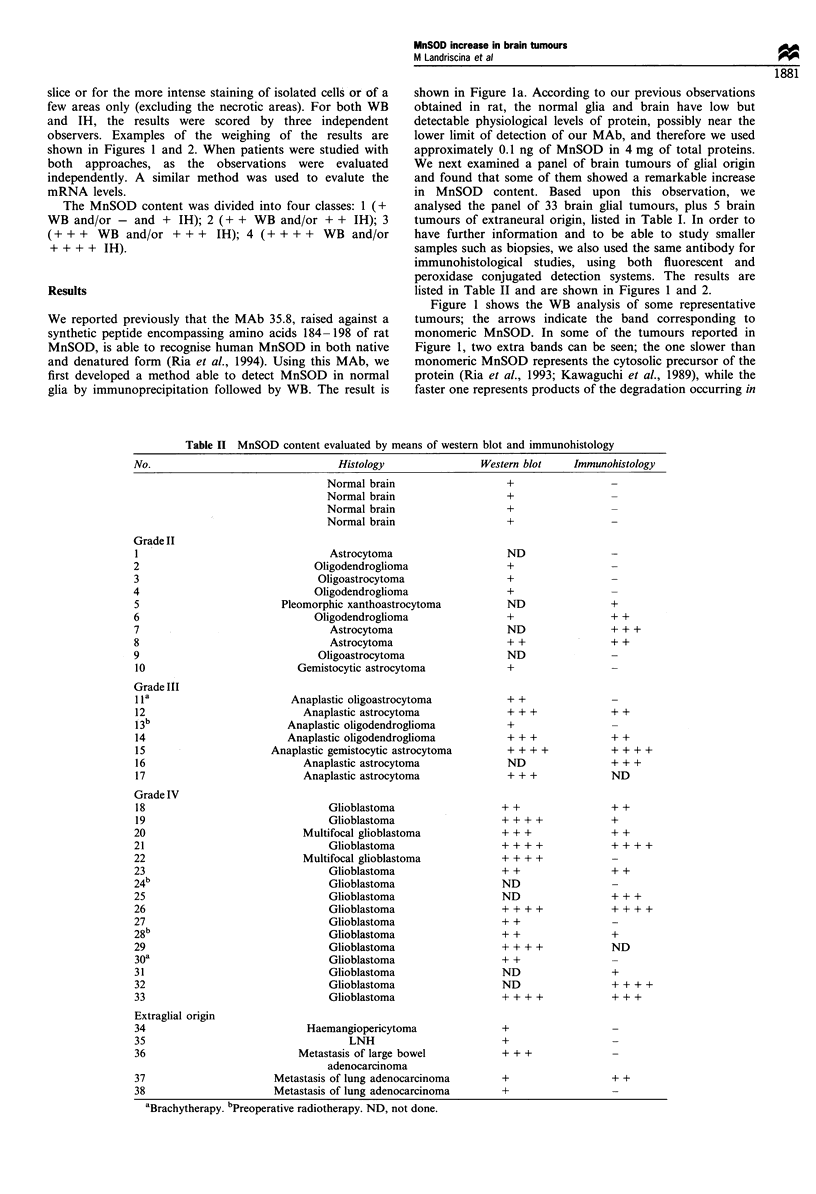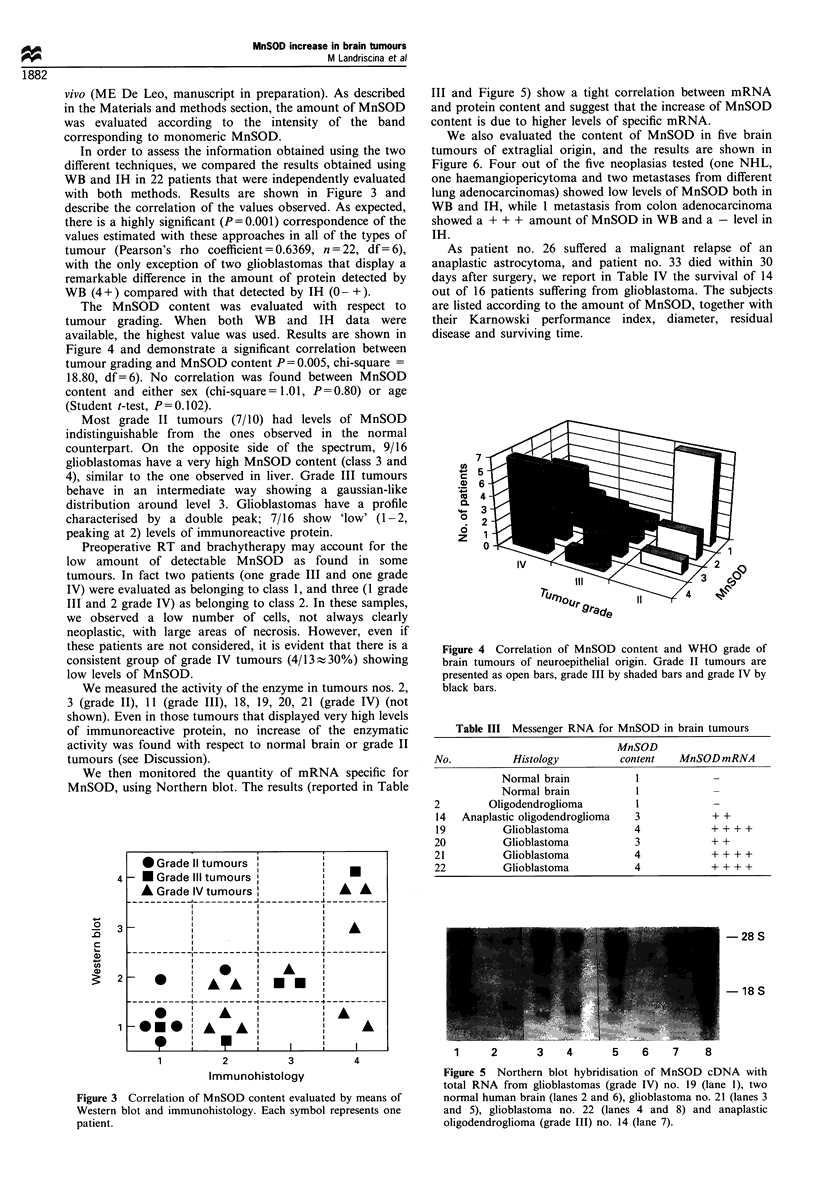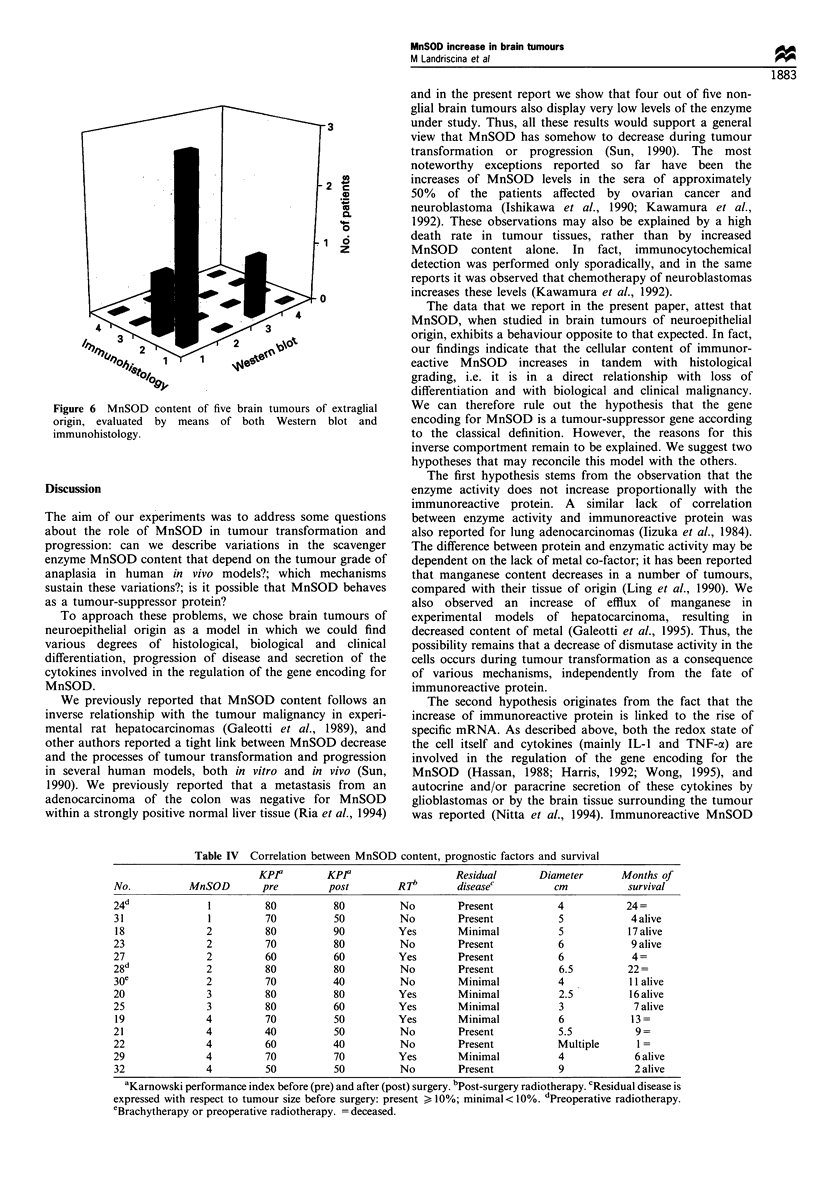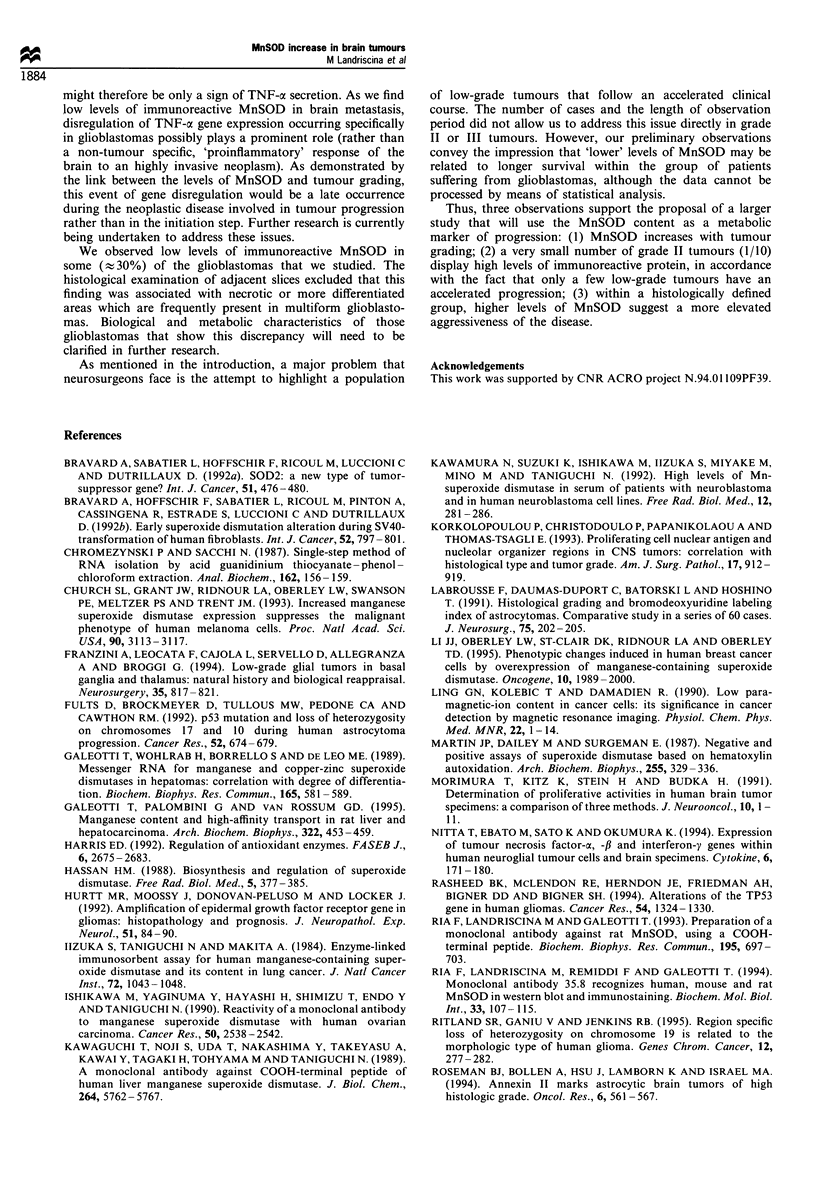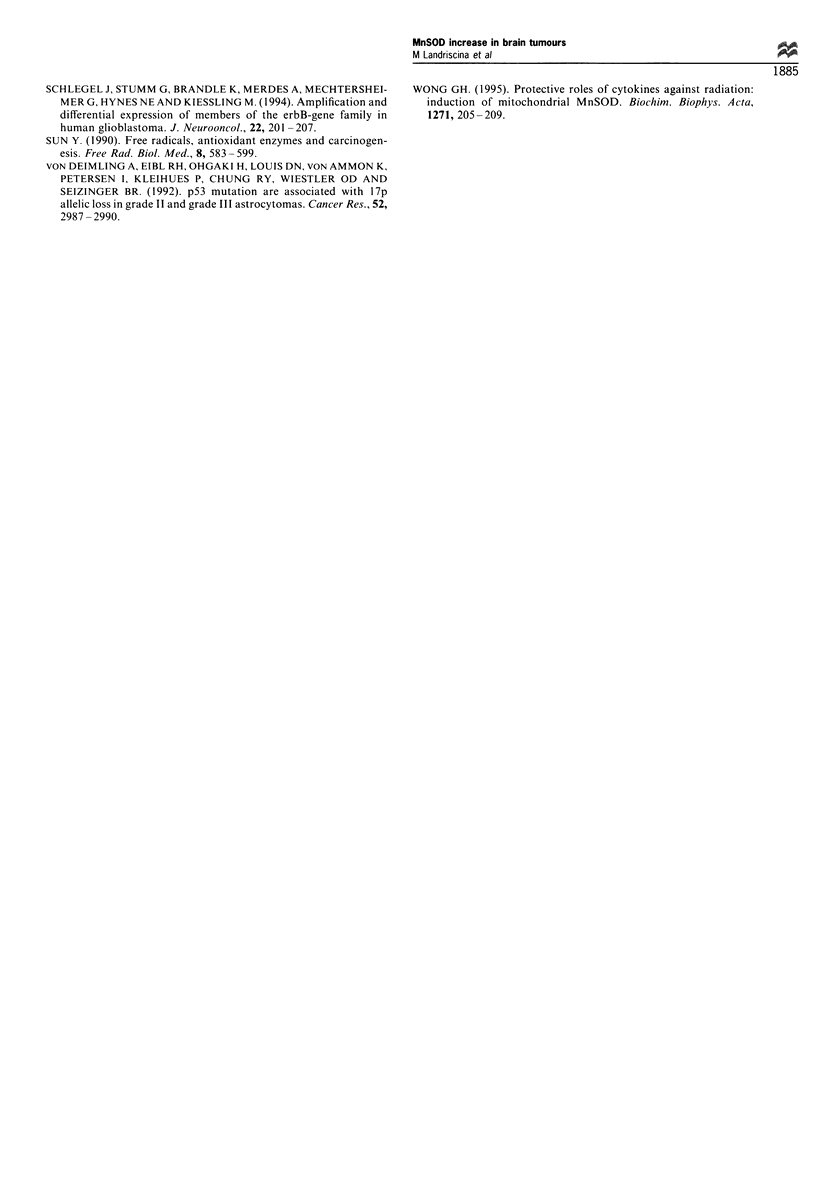# The level of MnSOD is directly correlated with grade of brain tumours of neuroepithelial origin.

**DOI:** 10.1038/bjc.1996.648

**Published:** 1996-12

**Authors:** M. Landriscina, F. Remiddi, F. Ria, B. Palazzotti, M. E. De Leo, M. Iacoangeli, R. Rosselli, M. Scerrati, T. Galeotti

**Affiliations:** Institute of General Pathology, Catholic University, Rome, Italy.

## Abstract

**Images:**


British Journal of Cancer (1996) 74, 1877–1885
© 1996 Stockton Press All rights reserved 0007–0920/96 $12.00

### The level of MnSOD is directly correlated with grade of brain tumours of neuroepithelial origin

M Landriscina[1], F Remiddi[1], F Ria[1], B Palazzotti[1], ME De Leo[1], M Iacoangeli[2], R Rosselli[2], M Scerrati[2] and T Galeotti[1]

[1]*Institute of General Pathology and* [2]*Neurosurgery, Catholic University, Largo F. Vito 1, 00168 Rome, Italy.*

**Summary** The oxy-radical scavenger enzyme manganese superoxide dismutase (MnSOD) may act in the capacity of a tumour-suppressor gene. To address the issue of its role in tumour transformation and progression *in vivo*, we evaluated the content of this enzyme in 33 brain tumours of neuroepithelial origin with different degrees of differentiation (WHO grade II–IV) by means of Western blot and immunohistology. Our results show that immunoreactive MnSOD increases in a direct relationship with tumour grade and is therefore inversely correlated with differentiation. The increase is induced at a pretranscriptional level and is apparently specific to brain tumours of neuroepithelial origin. Approximately 30% of grade IV tumours display low levels of MnSOD content, and preoperative radiotherapy and brachytherapy result in low amounts of enzyme. Based upon these observations, we suggest that MnSOD cannot be considered a classical tumour-suppressor gene.

**Keywords:** MnSOD; brain tumour; tumour grading; progression; tumour-suppressor gene

The mitochondrial scavenger enzyme manganese superoxide dismutase (MnSOD) is one of the three SODs that catalyse the dismutation of superoxide radicals to oxygen and hydrogen peroxide in eukaryotes, the other two being cytosolic CuZnSOD and extracellular CuZnSOD. While cytosolic CuZnSOD is a constitutive enzyme, MnSOD is an inducible protein, and the expression of its gene is regulated by the redox state of the cell and by cytokines, among which a dominant role is played by IL-1 and TNF-α (Hassan, 1988; Harris, 1992; Wong, 1995).

In experimental rat hepatocarcinomas, the MnSOD content decreases following an inverse relationship with the degree of differentiation (Galeotti *et al.*, 1989). A decrease in MnSOD content has also been reported in human neoplastic pathology, both in *in vitro* cell lines and in *in vivo* tumours (Sun, 1990). Furthermore, the steps of human hepatocarcinogenesis are accompanied by a progressive decrease of both CuZnSOD and MnSOD. According to these observations, a reduction in MnSOD has been looked at as a key point for malignant transformation and/or tumour progression, and it has been suggested that MnSOD could behave as a tumour-suppressor protein by preventing oxy-radical-mediated DNA damage (Sun, 1990; Bravard *et al.*, 1992*a*, *b*; Church *et al.*, 1993; Li *et al.*, 1995).

In contrast with this hypothesis, conflicting results have been reported for lung cancers (Iizuka *et al.*, 1984). Moreover, measurement of MnSOD by ELISA showed that its levels were increased in the sera of 12 out of 23 patients suffering from ovarian tumours (Ishikawa *et al.*, 1990) and in 8 out of 12 patients suffering from neuroblastoma (Kawamura *et al.*, 1992).

Brain tumours of neuroepithelial origin are classified on the basis of histological characteristics and of predominant cell type. Presence and degree of nuclear atypias, atypical mitosis, cell polymorphism, vascular proliferation and necrosis, together with the mitotic index, determine the histological grading. These tumours offer an interesting model for research, dealing with the processes of tumour initiation and progression. In most cases, the histological grade of malignancy is linked to their prognosis, as happens in experimental tumours. Better differentiated tumours often evolve into more malignant forms, demonstrating an example of progression of neoplastic disease. It has also been reported that TNF-α can be secreted by human glioblastoma cell lines, by some glioblastomas and by peritumoral brain tissue (Nitta *et al.*, 1994). Therefore, it was interesting to examine the role and regulation of MnSOD in the group of brain tumours of glial origin as they offer an *in vivo* model in which different degrees of differentiation, progression of disease and TNF-α secretion can be found.

A further point of interest in such a study is the fact that some low-grade tumours have an unexpectedly accelerated evolution towards high-grade, highly malignant tumours. One goal of current research in neurosurgery is to identify histological and/or biochemical parameters that may help in highlighting such a group within the good-prognosis, low-grade group of malignancies. This type of marker would allow identification of patients with less favourable prognosis who would benefit from more aggressive treatment. Several biological markers have been studied in this respect. Some of these markers are related to growth rate and cell cycle, such as labelling index (Franzini *et al.*, 1994), PCNA (Korkolopoulou *et al.*, 1993), BrdU (Labrousse *et al.*, 1991) and Ki-67 (Morimura *et al.*, 1991). Others refer more directly to tumour biology, such as amplification of the EGF receptor gene (Hurtt *et al.*, 1992; Schlegel *et al.*, 1994), loss of heterozygosity (LOH) for p53 (Fults *et al.*, 1992; von Deimling *et al.*, 1992; Rasheed *et al.*, 1994), LOH for chromosome 19q (Ritland *et al.*, 1995) and annexin II expression (Roseman *et al.*, 1994). However, the field is still open to further contributions as no marker has yet proved to be an effective identifier. Therefore, it would also make sense to evaluate in a preliminary way whether the MnSOD can be studied as a metabolic marker of progression. If so, it might be used in a study dedicated to prove its effectiveness in identifying the group of low-grade tumours that are at a higher risk of accelerated progression of the disease.

The main goal of the experiments that we report in the present paper was to further our knowledge on the role of MnSOD in tumour transformation and progression in an *in vivo* human model. We asked whether variation of MnSOD can be observed in tumours of glial origin with respect to normal controls, and if these variations are linked to the degree of differentiation and, if so, which molecular mechanism is responsible for them.

In order to address these issues, we analysed 38 brain tumours, 33 of neuroepithelial origin and five of extraneural origin. We assessed the amount of the MnSOD protein in the neoplastic tissues and evaluated the expression of its mRNA. These results were subsequently compared with the histological grade.

Correspondence: T Galeotti
Received 31 January 1996; revised 30 May 1996; accepted 28 June 1996

### Materials and methods

### *Patients*

Between May 1994 and October 1995, specimens from 33 brain tumours of neuroglial origin were collected (see Table I). Twenty-one patients were male (M) and 12 female (F); the mean age was 49.4 years (range 25–75 years). Sixteen tumours were glioblastomas (designated from now on as grade IV according to WHO classification) [M/F, 7/9; mean age 56.4 (range 32–75) years], four were anaplastic astrocytomas and the others included one anaplastic oligoastrocytoma and two anaplastic oligodendrogliomas (collectively referred to as grade III, WHO) [M/F, 7/1; mean age 40 (range 27–64) years], four astrocytomas, one pleiomorphic xanthoastrocytoma, three oligodendrogliomas and two oligoastrocytomas (hereafter grade II, WHO) [M/F, 7/2; mean age 42.4 (range 25–60) years]. All tumours were localised in the hemispherical white matter, and the intraoperative localisation of the tumours was obtained by means of ultrasonography.

In addition, samples from five brain tumours of extraglial origin (three metastasis, one non-Hodgkin lymphoma (NHL), one haemangiopericytoma) and four samples of tissue from normal, non-infiltrated areas were analysed. Five patients (three grade IV and two grade III) underwent either preoperative radiotherapy (RT) (3) or brachytherapy (2) shortly before the removal of tumour.

All the samples were intraoperative specimens, obtained during surgical removal of the brain tumour. They were divided into 125 $mm^3$ pieces and immediately frozen in liquid nitrogen and stored at −80°C. Samples were thawed only once and were analysed 15–30 days after collection; one piece was used for histology, one for Western blot (WB) analysis and one for immunohistology (IH) and 'mirror' conventional histology. For eight specimens (two normal cortexes, one grade II, one grade III and four grade IV tumours), an aliquot was used for RNA preparation.

The MnSOD studies were performed double blind with respect to histology, clinical status and degree of surgical removal.

Of the 33 samples from tumours of neuroepithelial origin, 22 were examined both by WB and IH, two by WB only and nine by IH alone.

### *Monoclonal antibody*

The preparation and maintenance of monoclonal antibody (MAb) 35.8 has previously been described (Ria *et al.*, 1993).

**Table I** Characteristics of patients

| *No.* | *Histology* | *Age* | *Sex* | *KPI*[a] *Pre* | *KPI*[a] *Post* | *Diameter cm* |
|---|---|---|---|---|---|---|
| Grade II | | | | | | |
| 1 | Astrocytoma | 44 | M | 80 | 80 | 9 |
| 2 | Oligodendroglioma | 46 | M | 90 | 80 | 4 |
| 3 | Oligoastrocytoma | 30 | M | 90 | 60 | 5 |
| 4 | Oligodendroglioma | 45 | M | 80 | 80 | 4 |
| 5 | Pleomorphic xanthoastrocytoma | 36 | M | 80 | 80 | 5 |
| 6 | Oligodendroglioma | 30 | M | 80 | 80 | 3.5 |
| 7 | Astrocytoma | 60 | M | 90 | 90 | 7 |
| 8 | Astrocytoma | 50 | F | 90 | 90 | 4.5 |
| 9 | Oligoastrocytoma | 47 | M | 90 | 90 | 5 |
| 10 | Gemistocytic astrocytoma | 25 | F | 90 | 90 | 6 |
| Grade III | | | | | | |
| 11[b] | Anaplastic oligoastrocytoma | 32 | M | 90 | 90 | 3.5 |
| 12 | Anaplastic astrocytoma | 45 | M | 90 | 90 | 3 |
| 13[c] | Anaplastic oligodendroglioma | 27 | F | 70 | 70 | 7 |
| 14 | Anaplastic oligodendroglioma | 46 | M | 70 | 70 | 5 |
| 15 | Anaplastic gemistocytic astrocytoma | 39 | M | 90 | 90 | 3.5 |
| 16 | Anaplastic astrocytoma | 64 | M | 50 | 60 | 8 |
| 17 | Anaplastic astrocytoma | 36 | M | 70 | 60 | 3.5 |
| Grade IV | | | | | | |
| 18 | Glioblastoma | 67 | M | 80 | 90 | 5 |
| 19 | Glioblastoma | 46 | M | 70 | 50 | 6 |
| 20 | Multifocal glioblastoma | 59 | F | 80 | 80 | 2.5 |
| 21 | Glioblastoma | 74 | F | 40 | 50 | 5.5 |
| 22 | Multifocal glioblastoma | 63 | F | 60 | 40 | |
| 23 | Glioblastoma | 53 | M | 70 | 80 | 6 |
| 24[c] | Glioblastoma | 55 | M | 80 | 80 | 4 |
| 25 | Glioblastoma | 45 | F | 80 | 60 | 3 |
| 26 | Glioblastoma | 32 | M | 80 | 80 | 5 |
| 27 | Glioblastoma | 57 | F | 60 | 60 | 6 |
| 28[c] | Glioblastoma | 45 | M | 80 | 80 | 6.5 |
| 29 | Glioblastoma | 49 | F | 70 | 70 | 4 |
| 30[b] | Glioblastoma | 57 | F | 70 | 40 | 4 |
| 31 | Glioblastoma | 66 | F | 70 | 50 | 5 |
| 32 | Glioblastoma | 59 | M | 50 | 50 | 9 |
| 33 | Glioblastoma | 75 | F | 80 | dec. | 4.8 |
| Extraglial origin | | | | | | |
| 34 | Haemangiopericytoma | 17 | F | 70 | 90 | 9 |
| 35 | NHL | 51 | F | 70 | 60 | 3.5 |
| 36 | Metastasis of large bowel adenocarcinoma | 61 | F | 80 | 80 | 4 |
| 37 | Metastasis of lung adenocarcinoma | 34 | F | 70 | 70 | 3.5 |
| 38 | Metastasis of lung adenocarcinoma | 57 | M | 40 | dec. | 9 |

[a]Karnowski performance index before (pre) and after (post) surgery. [b]Brachytherapy. [c]Preoperative radiotherapy. dec., Patient deceased.

We have also reported that this MAb is able to recognise human MnSOD in WB and immunofluorescence, and that it was able to detect 80 pg of human protein (Ria et al., 1994) in WB.

*Immunohistology*

Immediately before use, slices were cut and fixed in acetone–methanol (50%, v/v) for 10 min at 4°C. Slices were then washed three times by immersion in phosphate-buffered saline (PBS) 0.1 M for 30 min, followed by a 15 min incubation with protein blocking agent (Immunon-Lipshaw, Pittsburgh, PA, USA). After a fast wash in PBS (0.1 M), the preparations were incubated at room temperature with the supernatant of MAb 35.8, concentrated 50-fold by means of Minicon concentrators (Amicon) or with normal mouse serum diluted 1:80 in PBS (0.1 M) as control. Two hours later the slices were washed by immersion in 0.1 M PBS for 10 min, and incubated for 30 min with goat anti-mouse antibody conjugated with FITC (for immunofluorescence, Coulter) or with alkaline phosphatase (Sigma) diluted according to manufacturer's indications. After a final wash, BCIP/NBT (20 mg $ml^{-1}$ in water, Sigma) was added for 30 min. The reaction was stopped after 30 min by a very fast wash in water.

To exclude the possibility that the immuno-histological analysis had been performed on areas that were either more differentiated or necrotic, or even on peripheral normal brain, we re-evaluated the histology of slices sequential to the ones on which the immunostaining had been performed.

*Immunoprecipitation*

Tumour specimens were homogenised in potassium phosphate buffer (200 $\mu$g wet weight $ml^{-1}$) (as described in Galeotti et al., 1989, except in our case no dialysis was performed). Protein concentration was determined using the biuret method. The homogenates were then incubated with the supernatant of the MAb (250 $\mu$l $mg^{-1}$ protein for a total of 4 mg of protein) for 6 days at 4°C under continuous rolling. An aliquot of 30 $\mu$l of Sepharose conjugated with goat anti-mouse antiserum was added and the mixture was incubated at 37°C under continuous agitation. One hour later it was centrifuged for 10 min at 14 000 r.p.m., and the pellets recovered were resuspended in 15 $\mu$l of sample buffer for SDS-PAGE and WB analysis.

*Western blot*

The method for immunoblot detection of MnSOD has previously been described (Ria et al., 1993). In brief, SDS gel-electrophoresis was performed on a 12.5% gel. Immunoprecipitated samples were loaded and separated proteins were horizontally electroluted onto nitrocellulose paper. Immunoblot detection of MnSOD was performed with the supernatant of 35.8 hybridoma, seeded at $10^6$ cells $ml^{-1}$ and incubated at 37°C and 5% carbon dioxide for 30 days, using a phosphatase-labelled goat anti-mouse IgG antiserum (Sigma) as detection system. The amount of MnSOD was evaluated on the intensity of the band corresponding to

**Figure 1** Western blot of immunoprecipitates of homogenates from normal human liver, normal human brain and esemplificative tumours. The arrows indicate the band corresponding to monomeric MnSOD. Lane 1 in **a** and lane 6 in **b** show the 27.5 and 18.5 kDa MW standard (Biorad).

monomeric MnSOD (26 kDa apparent molecular weight). As a positive control, a sample immunoprecipitated from human normal liver was also run. This gave a reproducible intensity of the band corresponding to MnSOD. To exclude inter-sample variations, most samples were run at least twice.

*RNA isolation*

RNA was isolated as according to Chomezynski *et al.* (1987) by RNAs extraction and purification solution (Bioprobe). RNA concentrations were determined spectrophotometrically by absorbance at 260 nm.

*Quantitative analysis of mRNAs*

Total RNA for the analysis of MnSOD specific mRNA was size fractionated by formaldehyde–agarose (1.5%) gel electrophoresis and blotted onto nylon membrane. Blots were prehybridised for 5 h and hybridised to MnSOD cDNA overnight at 42°C. The cDNA probe was radiolabelled with [α-$^{32}$P]dCTP using a multiprime DNA labelling system (Amersham).

*Measurement of MnSOD activity*

The method published in Martin *et al.* (1987) was followed. In brief, SOD activity was assayed on the homogenised samples from the value giving 50% inhibition of haematoxylin autoxidation to haematein and was monitored at 560 nm and at pH 7.5 and 25°C, using a standard curve obtained with the purified bovine blood enzyme. MnSOD activity was measured in the presence of 1.5 mM cyanide.

*Interpretation of the results*

The results were expressed on a semiquantitative scale in comparison with human liver (+ + + + in both WB and IH) and normal glia (+ in WB and − in IH). In IH when evaluating the observation, intermediate values were given either for lower intensity of staining throughout the entire

**Figure 2** Immunohistochemical detection of MnSOD in tumours no. 3 (**a**) −, bar = 40 μM, 14 (**b**) (+ +, bar = 62.5 μM), 16 (**c**) (+ + +, bar = 62.5 μM) and 21 (**d**) (+ + + +, bar = 40 μM). (**e**) Immunostaining of the boundary region between tumour positive for MnSOD (+ + +) and peritumoral normal brain (patient 16). bar = 40 μM.

slice or for the more intense staining of isolated cells or of a few areas only (excluding the necrotic areas). For both WB and IH, the results were scored by three independent observers. Examples of the weighing of the results are shown in Figures 1 and 2. When patients were studied with both approaches, as the observations were evaluated independently. A similar method was used to evalute the mRNA levels.

The MnSOD content was divided into four classes: 1 (+ WB and/or − and + IH); 2 (+ + WB and/or + + IH); 3 (+ + + WB and/or + + + IH); 4 (+ + + + WB and/or + + + + IH).

### Results

We reported previously that the MAb 35.8, raised against a synthetic peptide encompassing amino acids 184–198 of rat MnSOD, is able to recognise human MnSOD in both native and denatured form (Ria *et al.*, 1994). Using this MAb, we first developed a method able to detect MnSOD in normal glia by immunoprecipitation followed by WB. The result is shown in Figure 1a. According to our previous observations obtained in rat, the normal glia and brain have low but detectable physiological levels of protein, possibly near the lower limit of detection of our MAb, and therefore we used approximately 0.1 ng of MnSOD in 4 mg of total proteins. We next examined a panel of brain tumours of glial origin and found that some of them showed a remarkable increase in MnSOD content. Based upon this observation, we analysed the panel of 33 brain glial tumours, plus 5 brain tumours of extraneural origin, listed in Table I. In order to have further information and to be able to study smaller samples such as biopsies, we also used the same antibody for immunohistological studies, using both fluorescent and peroxidase conjugated detection systems. The results are listed in Table II and are shown in Figures 1 and 2.

Figure 1 shows the WB analysis of some representative tumours; the arrows indicate the band corresponding to monomeric MnSOD. In some of the tumours reported in Figure 1, two extra bands can be seen; the one slower than monomeric MnSOD represents the cytosolic precursor of the protein (Ria *et al.*, 1993; Kawaguchi *et al.*, 1989), while the faster one represents products of the degradation occurring *in*

**Table II** MnSOD content evaluated by means of western blot and immunohistology

| *No.* | *Histology* | *Western blot* | *Immunohistology* |
|---|---|---|---|
| | Normal brain | + | − |
| | Normal brain | + | − |
| | Normal brain | + | − |
| | Normal brain | + | − |
| Grade II | | | |
| 1 | Astrocytoma | ND | − |
| 2 | Oligodendroglioma | + | − |
| 3 | Oligoastrocytoma | + | − |
| 4 | Oligodendroglioma | + | − |
| 5 | Pleomorphic xanthoastrocytoma | ND | + |
| 6 | Oligodendroglioma | + | + + |
| 7 | Astrocytoma | ND | + + + |
| 8 | Astrocytoma | + + | + + |
| 9 | Oligoastrocytoma | ND | − |
| 10 | Gemistocytic astrocytoma | + | − |
| Grade III | | | |
| 11[a] | Anaplastic oligoastrocytoma | + + | − |
| 12 | Anaplastic astrocytoma | + + + | + + |
| 13[b] | Anaplastic oligodendroglioma | + | − |
| 14 | Anaplastic oligodendroglioma | + + + | + + |
| 15 | Anaplastic gemistocytic astrocytoma | + + + + | + + + + |
| 16 | Anaplastic astrocytoma | ND | + + + |
| 17 | Anaplastic astrocytoma | + + + | ND |
| Grade IV | | | |
| 18 | Glioblastoma | + + | + + |
| 19 | Glioblastoma | + + + + | + |
| 20 | Multifocal glioblastoma | + + + | + + |
| 21 | Glioblastoma | + + + + | + + + + |
| 22 | Multifocal glioblastoma | + + + + | − |
| 23 | Glioblastoma | + + | + + |
| 24[b] | Glioblastoma | ND | − |
| 25 | Glioblastoma | ND | + + + |
| 26 | Glioblastoma | + + + + | + + + + |
| 27 | Glioblastoma | + + | − |
| 28[b] | Glioblastoma | + + | + |
| 29 | Glioblastoma | + + + + | ND |
| 30[a] | Glioblastoma | + + | − |
| 31 | Glioblastoma | ND | + |
| 32 | Glioblastoma | ND | + + + + |
| 33 | Glioblastoma | + + + + | + + + |
| Extraglial origin | | | |
| 34 | Haemangiopericytoma | + | − |
| 35 | LNH | + | − |
| 36 | Metastasis of large bowel adenocarcinoma | + + + | − |
| 37 | Metastasis of lung adenocarcinoma | + | + + |
| 38 | Metastasis of lung adenocarcinoma | + | − |

[a]Brachytherapy. [b]Preoperative radiotherapy. ND, not done.

*vivo* (ME De Leo, manuscript in preparation). As described in the Materials and methods section, the amount of MnSOD was evaluated according to the intensity of the band corresponding to monomeric MnSOD.

In order to assess the information obtained using the two different techniques, we compared the results obtained using WB and IH in 22 patients that were independently evaluated with both methods. Results are shown in Figure 3 and describe the correlation of the values observed. As expected, there is a highly significant ($P=0.001$) correspondence of the values estimated with these approaches in all of the types of tumour (Pearson's rho coefficient=0.6369, $n=22$, df=6), with the only exception of two glioblastomas that display a remarkable difference in the amount of protein detected by WB (4+) compared with that detected by IH (0−+).

The MnSOD content was evaluated with respect to tumour grading. When both WB and IH data were available, the highest value was used. Results are shown in Figure 4 and demonstrate a significant correlation between tumour grading and MnSOD content $P=0.005$, chi-square = 18.80, df=6). No correlation was found between MnSOD content and either sex (chi-square=1.01, $P=0.80$) or age (Student *t*-test, $P=0.102$).

Most grade II tumours (7/10) had levels of MnSOD indistinguishable from the ones observed in the normal counterpart. On the opposite side of the spectrum, 9/16 glioblastomas have a very high MnSOD content (class 3 and 4), similar to the one observed in liver. Grade III tumours behave in an intermediate way showing a gaussian-like distribution around level 3. Glioblastomas have a profile characterised by a double peak; 7/16 show 'low' (1−2, peaking at 2) levels of immunoreactive protein.

Preoperative RT and brachytherapy may account for the low amount of detectable MnSOD as found in some tumours. In fact two patients (one grade III and one grade IV) were evaluated as belonging to class 1, and three (1 grade III and 2 grade IV) as belonging to class 2. In these samples, we observed a low number of cells, not always clearly neoplastic, with large areas of necrosis. However, even if these patients are not considered, it is evident that there is a consistent group of grade IV tumours (4/13≈30%) showing low levels of MnSOD.

We measured the activity of the enzyme in tumours nos. 2, 3 (grade II), 11 (grade III), 18, 19, 20, 21 (grade IV) (not shown). Even in those tumours that displayed very high levels of immunoreactive protein, no increase of the enzymatic activity was found with respect to normal brain or grade II tumours (see Discussion).

We then monitored the quantity of mRNA specific for MnSOD, using Northern blot. The results (reported in Table III and Figure 5) show a tight correlation between mRNA and protein content and suggest that the increase of MnSOD content is due to higher levels of specific mRNA.

We also evaluated the content of MnSOD in five brain tumours of extraglial origin, and the results are shown in Figure 6. Four out of the five neoplasias tested (one NHL, one haemangiopericytoma and two metastases from different lung adenocarcinomas) showed low levels of MnSOD both in WB and IH, while 1 metastasis from colon adenocarcinoma showed a + + + amount of MnSOD in WB and a − level in IH.

As patient no. 26 suffered a malignant relapse of an anaplastic astrocytoma, and patient no. 33 died within 30 days after surgery, we report in Table IV the survival of 14 out of 16 patients suffering from glioblastoma. The subjects are listed according to the amount of MnSOD, together with their Karnowski performance index, diameter, residual disease and surviving time.

**Figure 3** Correlation of MnSOD content evaluated by means of Western blot and immunohistology. Each symbol represents one patient.

**Figure 4** Correlation of MnSOD content and WHO grade of brain tumours of neuroepithelial origin. Grade II tumours are presented as open bars, grade III by shaded bars and grade IV by black bars.

**Table III** Messenger RNA for MnSOD in brain tumours

| *No.* | *Histology* | *MnSOD content* | *MnSOD mRNA* |
|---|---|---|---|
| | Normal brain | 1 | − |
| | Normal brain | 1 | − |
| 2 | Oligodendroglioma | 1 | − |
| 14 | Anaplastic oligodendroglioma | 3 | + + |
| 19 | Glioblastoma | 4 | + + + + |
| 20 | Glioblastoma | 3 | + + |
| 21 | Glioblastoma | 4 | + + + + |
| 22 | Glioblastoma | 4 | + + + + |

**Figure 5** Northern blot hybridisation of MnSOD cDNA with total RNA from glioblastomas (grade IV) no. 19 (lane 1), two normal human brain (lanes 2 and 6), glioblastoma no. 21 (lanes 3 and 5), glioblastoma no. 22 (lanes 4 and 8) and anaplastic oligodendroglioma (grade III) no. 14 (lane 7).

**Figure 6** MnSOD content of five brain tumours of extraglial origin, evaluated by means of both Western blot and immunohistology.

### Discussion

The aim of our experiments was to address some questions about the role of MnSOD in tumour transformation and progression: can we describe variations in the scavenger enzyme MnSOD content that depend on the tumour grade of anaplasia in human *in vivo* models?; which mechanisms sustain these variations?; is it possible that MnSOD behaves as a tumour-suppressor protein?

To approach these problems, we chose brain tumours of neuroepithelial origin as a model in which we could find various degrees of histological, biological and clinical differentiation, progression of disease and secretion of the cytokines involved in the regulation of the gene encoding for MnSOD.

We previously reported that MnSOD content follows an inverse relationship with the tumour malignancy in experimental rat hepatocarcinomas (Galeotti *et al.*, 1989), and other authors reported a tight link between MnSOD decrease and the processes of tumour transformation and progression in several human models, both *in vitro* and *in vivo* (Sun, 1990). We previously reported that a metastasis from an adenocarcinoma of the colon was negative for MnSOD within a strongly positive normal liver tissue (Ria *et al.*, 1994) and in the present report we show that four out of five non-glial brain tumours also display very low levels of the enzyme under study. Thus, all these results would support a general view that MnSOD has somehow to decrease during tumour transformation or progression (Sun, 1990). The most noteworthy exceptions reported so far have been the increases of MnSOD levels in the sera of approximately 50% of the patients affected by ovarian cancer and neuroblastoma (Ishikawa *et al.*, 1990; Kawamura *et al.*, 1992). These observations may also be explained by a high death rate in tumour tissues, rather than by increased MnSOD content alone. In fact, immunocytochemical detection was performed only sporadically, and in the same reports it was observed that chemotherapy of neuroblastomas increases these levels (Kawamura *et al.*, 1992).

The data that we report in the present paper, attest that MnSOD, when studied in brain tumours of neuroepithelial origin, exhibits a behaviour opposite to that expected. In fact, our findings indicate that the cellular content of immunoreactive MnSOD increases in tandem with histological grading, i.e. it is in a direct relationship with loss of differentiation and with biological and clinical malignancy. We can therefore rule out the hypothesis that the gene encoding for MnSOD is a tumour-suppressor gene according to the classical definition. However, the reasons for this inverse comportment remain to be explained. We suggest two hypotheses that may reconcile this model with the others.

The first hypothesis stems from the observation that the enzyme activity does not increase proportionally with the immunoreactive protein. A similar lack of correlation between enzyme activity and immunoreactive protein was also reported for lung adenocarcinomas (Iizuka *et al.*, 1984). The difference between protein and enzymatic activity may be dependent on the lack of metal co-factor; it has been reported that manganese content decreases in a number of tumours, compared with their tissue of origin (Ling *et al.*, 1990). We also observed an increase of efflux of manganese in experimental models of hepatocarcinoma, resulting in decreased content of metal (Galeotti *et al.*, 1995). Thus, the possibility remains that a decrease of dismutase activity in the cells occurs during tumour transformation as a consequence of various mechanisms, independently from the fate of immunoreactive protein.

The second hypothesis originates from the fact that the increase of immunoreactive protein is linked to the rise of specific mRNA. As described above, both the redox state of the cell itself and cytokines (mainly IL-1 and TNF-α) are involved in the regulation of the gene encoding for the MnSOD (Hassan, 1988; Harris, 1992; Wong, 1995), and autocrine and/or paracrine secretion of these cytokines by glioblastomas or by the brain tissue surrounding the tumour was reported (Nitta *et al.*, 1994). Immunoreactive MnSOD

**Table IV** Correlation between MnSOD content, prognostic factors and survival

| *No.* | *MnSOD* | *KPI[a] pre* | *KPI[a] post* | *RT[b]* | *Residual disease[c]* | *Diameter cm* | *Months of survival* |
|---|---|---|---|---|---|---|---|
| 24[d] | 1 | 80 | 80 | No | Present | 4 | 24 = |
| 31 | 1 | 70 | 50 | No | Present | 5 | 4 alive |
| 18 | 2 | 80 | 90 | Yes | Minimal | 5 | 17 alive |
| 23 | 2 | 70 | 80 | No | Present | 6 | 9 alive |
| 27 | 2 | 60 | 60 | Yes | Present | 6 | 4 = |
| 28[d] | 2 | 80 | 80 | No | Present | 6.5 | 22 = |
| 30[e] | 2 | 70 | 40 | No | Minimal | 4 | 11 alive |
| 20 | 3 | 80 | 80 | Yes | Minimal | 2.5 | 16 alive |
| 25 | 3 | 80 | 60 | Yes | Minimal | 3 | 7 alive |
| 19 | 4 | 70 | 50 | Yes | Minimal | 6 | 13 = |
| 21 | 4 | 40 | 50 | No | Present | 5.5 | 9 = |
| 22 | 4 | 60 | 40 | No | Present | Multiple | 1 = |
| 29 | 4 | 70 | 70 | Yes | Minimal | 4 | 6 alive |
| 32 | 4 | 50 | 50 | No | Present | 9 | 2 alive |

[a]Karnowski performance index before (pre) and after (post) surgery. [b]Post-surgery radiotherapy. [c]Residual disease is expressed with respect to tumour size before surgery: present ⩾10%; minimal < 10%. [d]Preoperative radiotherapy. [e]Brachytherapy or preoperative radiotherapy. = deceased.

might therefore be only a sign of TNF-α secretion. As we find low levels of immunoreactive MnSOD in brain metastasis, disregulation of TNF-α gene expression occurring specifically in glioblastomas possibly plays a prominent role (rather than a non-tumour specific, 'proinflammatory' response of the brain to an highly invasive neoplasm). As demonstrated by the link between the levels of MnSOD and tumour grading, this event of gene disregulation would be a late occurrence during the neoplastic disease involved in tumour progression rather than in the initiation step. Further research is currently being undertaken to address these issues.

We observed low levels of immunoreactive MnSOD in some (≈30%) of the glioblastomas that we studied. The histological examination of adjacent slices excluded that this finding was associated with necrotic or more differentiated areas which are frequently present in multiform glioblastomas. Biological and metabolic characteristics of those glioblastomas that show this discrepancy will need to be clarified in further research.

As mentioned in the introduction, a major problem that neurosurgeons face is the attempt to highlight a population of low-grade tumours that follow an accelerated clinical course. The number of cases and the length of observation period did not allow us to address this issue directly in grade II or III tumours. However, our preliminary observations convey the impression that 'lower' levels of MnSOD may be related to longer survival within the group of patients suffering from glioblastomas, although the data cannot be processed by means of statistical analysis.

Thus, three observations support the proposal of a larger study that will use the MnSOD content as a metabolic marker of progression: (1) MnSOD increases with tumour grading; (2) a very small number of grade II tumours (1/10) display high levels of immunoreactive protein, in accordance with the fact that only a few low-grade tumours have an accelerated progression; (3) within a histologically defined group, higher levels of MnSOD suggest a more elevated aggressiveness of the disease.

**Acknowledgements**
This work was supported by CNR ACRO project N.94.01109PF39.

### References

BRAVARD A, SABATIER L, HOFFSCHIR F, RICOUL M, LUCCIONI C AND DUTRILLAUX D. (1992*a*). SOD2: a new type of tumor-suppressor gene? *Int. J. Cancer*, **51**, 476–480.

BRAVARD A, HOFFSCHIR F, SABATIER L, RICOUL M, PINTON A, CASSINGENA R, ESTRADE S, LUCCIONI C AND DUTRILLAUX D. (1992*b*). Early superoxide dismutation alteration during SV40-transformation of human fibroblasts. *Int. J. Cancer*, **52**, 797–801.

CHROMEZYNSKI P AND SACCHI N. (1987). Single-step method of RNA isolation by acid guanidinium thiocyanate–phenol–chloroform extraction. *Anal. Biochem.*, **162**, 156–159.

CHURCH SL, GRANT JW, RIDNOUR LA, OBERLEY LW, SWANSON PE, MELTZER PS AND TRENT JM. (1993). Increased manganese superoxide dismutase expression suppresses the malignant phenotype of human melanoma cells. *Proc. Natl Acad. Sci. USA*, **90**, 3113–3117.

FRANZINI A, LEOCATA F, CAJOLA L, SERVELLO D, ALLEGRANZA A AND BROGGI G. (1994). Low-grade glial tumors in basal ganglia and thalamus: natural history and biological reappraisal. *Neurosurgery*, **35**, 817–821.

FULTS D, BROCKMEYER D, TULLOUS MW, PEDONE CA AND CAWTHON RM. (1992). p53 mutation and loss of heterozygosity on chromosomes 17 and 10 during human astrocytoma progression. *Cancer Res.*, **52**, 674–679.

GALEOTTI T, WOHLRAB H, BORRELLO S AND DE LEO ME. (1989). Messenger RNA for manganese and copper-zinc superoxide dismutases in hepatomas: correlation with degree of differentiation. *Biochem. Biophys. Res. Commun.*, **165**, 581–589.

GALEOTTI T, PALOMBINI G AND VAN ROSSUM GD. (1995). Manganese content and high-affinity transport in rat liver and hepatocarcinoma. *Arch. Biochem. Biophys.*, **322**, 453–459.

HARRIS ED. (1992). Regulation of antioxidant enzymes. *FASEB J.*, **6**, 2675–2683.

HASSAN HM. (1988). Biosynthesis and regulation of superoxide dismutase. *Free Rad. Biol. Med.*, **5**, 377–385.

HURTT MR, MOOSSY J, DONOVAN-PELUSO M AND LOCKER J. (1992). Amplification of epidermal growth factor receptor gene in gliomas: histopathology and prognosis. *J. Neuropathol. Exp. Neurol.*, **51**, 84–90.

IIZUKA S, TANIGUCHI N AND MAKITA A. (1984). Enzyme-linked immunosorbent assay for human manganese-containing superoxide dismutase and its content in lung cancer. *J. Natl Cancer Inst.*, **72**, 1043–1048.

ISHIKAWA M, YAGINUMA Y, HAYASHI H, SHIMIZU T, ENDO Y AND TANIGUCHI N. (1990). Reactivity of a monoclonal antibody to manganese superoxide dismutase with human ovarian carcinoma. *Cancer Res.*, **50**, 2538–2542.

KAWAGUCHI T, NOJI S, UDA T, NAKASHIMA Y, TAKEYASU A, KAWAI Y, TAGAKI H, TOHYAMA M AND TANIGUCHI N. (1989). A monoclonal antibody against COOH-terminal peptide of human liver manganese superoxide dismutase. *J. Biol. Chem.*, **264**, 5762–5767.

KAWAMURA N, SUZUKI K, ISHIKAWA M, IIZUKA S, MIYAKE M, MINO M AND TANIGUCHI N. (1992). High levels of Mn-superoxide dismutase in serum of patients with neuroblastoma and in human neuroblastoma cell lines. *Free Rad. Biol. Med.*, **12**, 281–286.

KORKOLOPOULOU P, CHRISTODOULO P, PAPANIKOLAOU A AND THOMAS-TSAGLI E. (1993). Proliferating cell nuclear antigen and nucleolar organizer regions in CNS tumors: correlation with histological type and tumor grade. *Am. J. Surg. Pathol.*, **17**, 912–919.

LABROUSSE F, DAUMAS-DUPORT C, BATORSKI L AND HOSHINO T. (1991). Histological grading and bromodeoxyuridine labeling index of astrocytomas. Comparative study in a series of 60 cases. *J. Neurosurg.*, **75**, 202–205.

LI JJ, OBERLEY LW, ST-CLAIR DK, RIDNOUR LA AND OBERLEY TD. (1995). Phenotypic changes induced in human breast cancer cells by overexpression of manganese-containing superoxide dismutase. *Oncogene*, **10**, 1989–2000.

LING GN, KOLEBIC T AND DAMADIEN R. (1990). Low paramagnetic-ion content in cancer cells: its significance in cancer detection by magnetic resonance imaging. *Physiol. Chem. Phys. Med. MNR*, **22**, 1–14.

MARTIN JP, DAILEY M AND SURGEMAN E. (1987). Negative and positive assays of superoxide dismutase based on hematoxylin autoxidation. *Arch. Biochem. Biophys.*, **255**, 329–336.

MORIMURA T, KITZ K, STEIN H AND BUDKA H. (1991). Determination of proliferative activities in human brain tumor specimens: a comparison of three methods. *J. Neurooncol.*, **10**, 1–11.

NITTA T, EBATO M, SATO K AND OKUMURA K. (1994). Expression of tumour necrosis factor-α, -β and interferon-γ genes within human neuroglial tumour cells and brain specimens. *Cytokine*, **6**, 171–180.

RASHEED BK, MCLENDON RE, HERNDON JE, FRIEDMAN AH, BIGNER DD AND BIGNER SH. (1994). Alterations of the TP53 gene in human gliomas. *Cancer Res.*, **54**, 1324–1330.

RIA F, LANDRISCINA M AND GALEOTTI T. (1993). Preparation of a monoclonal antibody against rat MnSOD, using a COOH-terminal peptide. *Biochem. Biophys. Res. Commun.*, **195**, 697–703.

RIA F, LANDRISCINA M, REMIDDI F AND GALEOTTI T. (1994). Monoclonal antibody 35.8 recognizes human, mouse and rat MnSOD in western blot and immunostaining. *Biochem. Mol. Biol. Int.*, **33**, 107–115.

RITLAND SR, GANIU V AND JENKINS RB. (1995). Region specific loss of heterozygosity on chromosome 19 is related to the morphologic type of human glioma. *Genes Chrom. Cancer*, **12**, 277–282.

ROSEMAN BJ, BOLLEN A, HSU J, LAMBORN K AND ISRAEL MA. (1994). Annexin II marks astrocytic brain tumors of high histologic grade. *Oncol. Res.*, **6**, 561–567.

SCHLEGEL J, STUMM G, BRANDLE K, MERDES A, MECHTERSHEIMER G, HYNES NE AND KIESSLING M. (1994). Amplification and differential expression of members of the erbB-gene family in human glioblastoma. *J. Neurooncol.*, **22,** 201-207.

SUN Y. (1990). Free radicals, antioxidant enzymes and carcinogenesis. *Free Rad. Biol. Med.*, **8,** 583-599.

VON DEIMLING A, EIBL RH, OHGAKI H, LOUIS DN, VON AMMON K, PETERSEN I, KLEIHUES P, CHUNG RY, WIESTLER OD AND SEIZINGER BR. (1992). p53 mutation are associated with 17p allelic loss in grade II and grade III astrocytomas. *Cancer Res.*, **52,** 2987-2990.

WONG GH. (1995). Protective roles of cytokines against radiation: induction of mitochondrial MnSOD. *Biochim. Biophys. Acta*, **1271,** 205-209.